# An agent-based model of leukocyte transendothelial migration during atherogenesis

**DOI:** 10.1371/journal.pcbi.1005523

**Published:** 2017-05-25

**Authors:** Rita Bhui, Heather N. Hayenga

**Affiliations:** 1 Department of Physics, University of Texas, Dallas, Richardson TX, United States of America; 2 Department of Bioengineering, University of Texas, Dallas, Richardson TX, United States of America; Stanford University, UNITED STATES

## Abstract

A vast amount of work has been dedicated to the effects of hemodynamics and cytokines on leukocyte adhesion and trans-endothelial migration (TEM) and subsequent accumulation of leukocyte-derived foam cells in the artery wall. However, a comprehensive mechanobiological model to capture these spatiotemporal events and predict the growth and remodeling of an atherosclerotic artery is still lacking. Here, we present a multiscale model of leukocyte TEM and plaque evolution in the left anterior descending (LAD) coronary artery. The approach integrates cellular behaviors via agent-based modeling (ABM) and hemodynamic effects via computational fluid dynamics (CFD). In this computational framework, the ABM implements the diffusion kinetics of key biological proteins, namely Low Density Lipoprotein (LDL), Tissue Necrosis Factor alpha (TNF-α), Interlukin-10 (IL-10) and Interlukin-1 beta (IL-1β), to predict chemotactic driven leukocyte migration into and within the artery wall. The ABM also considers wall shear stress (WSS) dependent leukocyte TEM and compensatory arterial remodeling obeying Glagov’s phenomenon. Interestingly, using fully developed steady blood flow does not result in a representative number of leukocyte TEM as compared to pulsatile flow, whereas passing WSS at peak systole of the pulsatile flow waveform does. Moreover, using the model, we have found leukocyte TEM increases monotonically with decreases in luminal volume. At critical plaque shapes the WSS changes rapidly resulting in sudden increases in leukocyte TEM suggesting lumen volumes that will give rise to rapid plaque growth rates if left untreated. Overall this multi-scale and multi-physics approach appropriately captures and integrates the spatiotemporal events occurring at the cellular level in order to predict leukocyte transmigration and plaque evolution.

## Author summary

Atherosclerosis affects millions of people worldwide and is characterized by a maladaptive build-up of fatty material, leukocytes, and extracellular matrix inside the artery wall. With age this material, collectively called a plaque, enhances and blocks blood flow thereby altering the hemodynamics. If the plaque ruptures, the occlusion may cause a life-threating stroke or myocardial infarction. Although it is known that local biochemical and hemodynamics influence leukocyte adhesion and trans-endothelial migration into the wall, their effects on the growth rates of plaques are less known. Being able to identify crucial plaque shapes that are susceptible to rapid growth rates if left untreated will help treat plaques vulnerable to rupturing. Therefore, we developed an agent-based model capable of integrating cell behaviors and hemodynamic properties as the plaque grows, and identified crucial lumen volumes that give rise to favorable conditions for rapid plaque growth. Collectively, understanding how mechanobiological events are integrated within an artery will help elucidate emergent behaviors and predict plaque evolution.

## Introduction

Cardiovascular Diseases (CVD) are still the leading cause of death in the United States. The most common cause of CVD is atherosclerosis [1]. Atherosclerosis is a local inflammatory disease characterized initially by the recruitment of leukocytes into the arterial wall. Through a cascade of events the arterial wall may develop a plaque, comprising of leukocyte-derived foam cells, lipids, calcium and other constituents [2]. When an atherosclerotic plaque ruptures, it may block blood flow completely, which results in a possibly life-threating stroke or myocardial infarction. Indeed, the less stenotic plaques may be more vulnerable due to their underlying structure. According to a metastudy approximately 85% of acute myocardial infarctions arose from lesions with degrees of stenosis less than 60% on an antecedent angiogram [3]. Leukocyte adhesion to, and transmigration through, the endothelium of blood vessels is an essential event in inflammation and the pathogenesis of atherosclerosis. The recruitment steps involved in the leukocyte transmigration cascade (i.e., capture, rolling, activation and adhesion) are well established [4–6]; however, a model which can predict the effects of these events is lacking. Hence we present herein an agent based model (ABM) for simulating the complex behavior of discrete autonomous agents (i.e. cells) in order to assess their effects on the artery and to deepen the understanding of the pathophysiology of atherogenesis.

The endothelium of an artery is in direct contact with the flowing blood and hence it is constantly exposed to the mechanical forces exerted by the blood. The frictional force per unit area of the vessel wall, called wall shear stress (WSS), is a critical factor in maintaining endothelial function and it varies over each cardiac cycle. Clinically hemodynamics has been used to predict areas of plaque progression [7–10] or the need for treatment (e.g., Fractional Flow Reserve). Experimentally, the dynamics relating WSS, endothelial cell activation and leukocyte trans-endothelial migration (TEM) have been quantified [11–13]. However, a computational model incorporating the interdependency of leukocyte TEM, plaque growth and WSS with time is lacking. Since, blood flow through an artery is pulsatile in nature, the WSS and therefore rate of leukocyte TEM vary with time. Using average WSS is not physiological as it does not capture the effect of low WSS as well as high WSS in TEM. Herein, we establish a time interval at which WSS is passed from the CFD model to the ABM in order to emulate pulsatile flow and create a model that incorporates WSS dependencies of plaque growth and remodeling.

In 1987 Glagov et al. reported the pivotal finding that atherosclerotic arteries initially remodel outward in attempt to preserve the luminal blood flow. Glagov and colleagues found that the external diameter increased while the lumen area of atherosclerotic human coronaries remained constant until the percent of plaque area exceeded 40% of the luminal area [14]. Although, the mechanisms for this compensatory remodeling effect are still being established [15], the Glagov phenomenon informs growth and remodeling (G&R) in our ABM of plaque evolution. Hence, we present a novel predictive model of leukocyte TEM by incorporating pathophysiological dependencies on changes to the mechanical (hemodynamics) and biological (leukocyte TEM, chemotaxis, outward remodeling, monocyte to foam cell differentiation, cell activation, and protein diffusion) environment to determine the evolution of an atherosclerotic plaque.

## Methods

### ABM design

**Fig 1:**
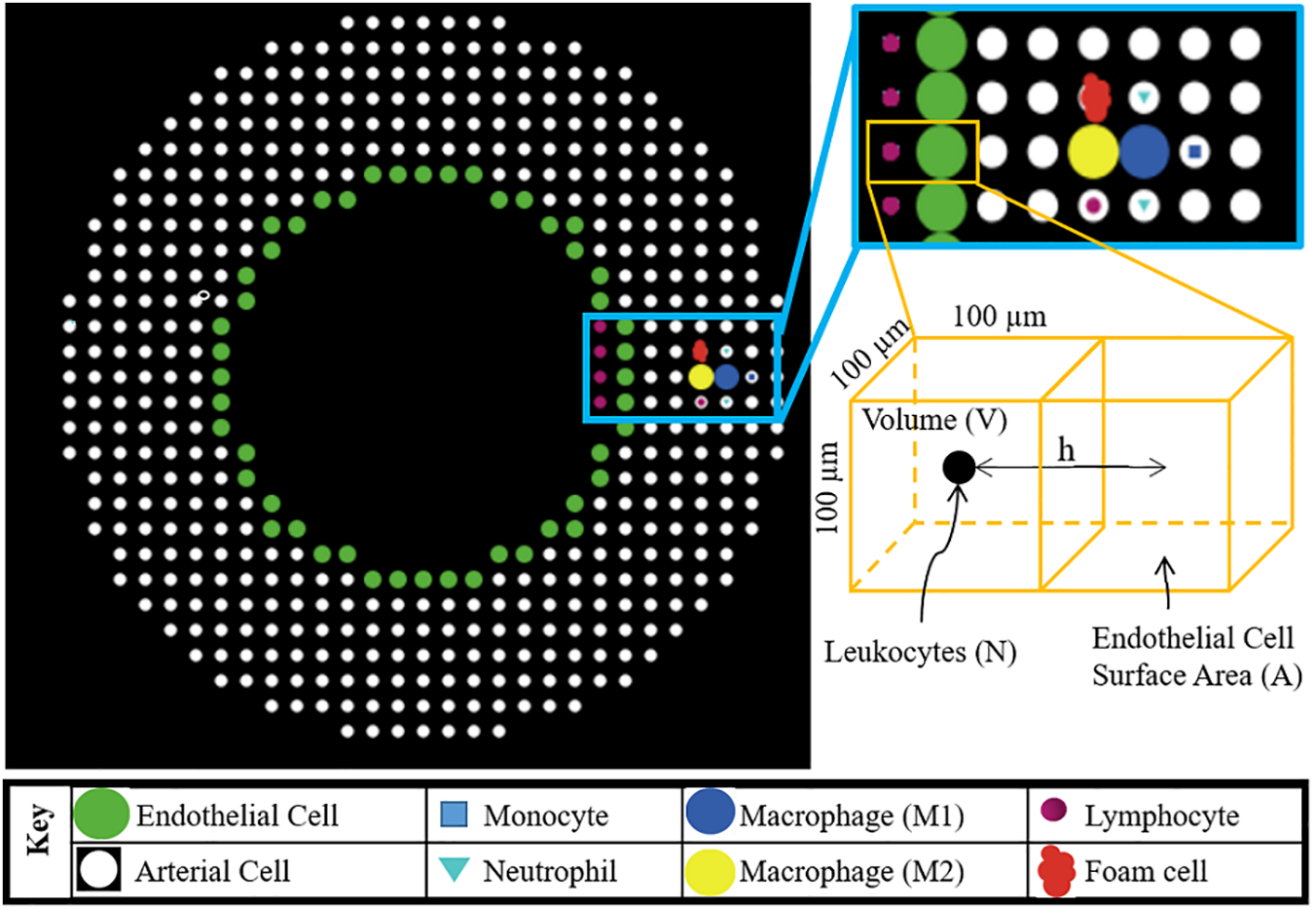
Schematic illustrating the spatial distribution of each type of cell (i.e. agent) in the ABM. The bottom right schematic represents a luminal and artery wall patch where N is the number of leukocyte agents residing in this luminal patch. Among all leukocytes (N), M is the number that adheres to the endothelial cell surface area (A). V is the patch volume and h is 100μm or the distance between the patch centroids. All these parameters are used to find leukocyte adhesion probability (see Eq 2).

Agent-based modeling is a class of computational modeling that predicts the evolution of a dynamical system by simulating the behavior of autonomous cellular components, called “agents.” The agents follow behavioral “rules” that control the responses to changes in their environmental or internal properties. This dynamic system allows for complex phenomenon to emerge from the interaction of simple rule-based behaviors of agents. The system (Fig 1) resides in a regularized 3D environment, commonly implemented as a grid. The grid spaces are called “patches” in the ABM and within each patch, the agents, extracellular matrix (ECM) and soluble factors can reside.

### System description

The 3D model of leukocyte TEM is developed in an open source ABM software, NetLogo 3D 5.3.1. The entire 3D space is segmented into cubical patches of edge length 100 μm. Fig 1 illustrates an arterial cross-section and all the agents included in this ABM. The modeled artery is allowed to spatiotemporally evolve whereby each time tick represents a 1-hour interval. The initial conditions and parameters are given in Table 1. Rules quantifying the influence of primary factors on leukocyte adhesion, TEM and plaque progression are derived from the published literature and are listed in Table 2.

**Table 1:** Initial ABM parameters.

Parameters	Value	References
Patch size (μm x μm x μm)	100x100x100	
One tick	One hour	
Lumen radius (μm)	1800	[16]
Artery length (μm)	6000	
Artery wall thickness (Media + Adventitia) (μm)	600	[17, 18]
Initial plaque	15 leukocytes	
Leukocyte concentration in blood	7x10^9^/liter	[19]
Neutrophil concentration in blood	62% of leukocytes	*
Monocyte concentration in blood	5.3% of leukocytes	*
Lymphocyte concentration in blood	30% of leukocytes	*
LDL concentration in blood	Normal: 950–970 ng/μl Unhealthy:1900–2000 ng/μl	[20]
Diffusion coefficient of cytokines	3x10^11^ m^2^/s	[21]
Diffusion coefficient of LDL	2.5x10^11^ m^2^/s	[22]
Healthy WSS (Pa)	1.4	[10]
Stiffness of artery wall (kPa)	Normal:3; Unhealthy: > 3	[23]
Diameter of neutrophils & lymphocytes (μm)	12	**
Diameter of monocytes (μm)	24	**
Diameter of foam cell (μm)	25	**

*Assigned values; patient specific

**Assigned values

**Table 2:** List of rules used in the ABM.

No.	Behavior	ABM Rule	Ref.
1.	Dependence of neutrophil adhesion on TNF-α	$\begin{array}{l} \frac{R}{\rho t}=\frac{1}{180}\left[0.80-0.68(1-exp(-0.33{\left({log}_{10}x\right)}^{-0.56}))\right];x>1\\ \;=\frac{1}{180}[0.0996x];x\leq 1 \end{array}$ *x*: *TNFα*[*U*/*ml*]; *R*[#/*mm*^2^]; *ρ*[#/*mm*^3^]; *t*[*sec*]	[24]
2.	Dependence of neutrophil adhesion on IL-1	$\frac{R}{\rho t}=\frac{1}{900}[0.10+0.19(1-exp(-28.29x^{1.46}))]$ *x*: *IL*1*β*[*U*/*ml*]; *R*[#/*mm*^2^]; *ρ*[#/*mm*^3^]; *t*[*sec*]	[25]
3.	Dependence of neutrophil adhesion on WSS	$\frac{R}{\rho t}=\left\{ \begin{array}{c} \frac{1}{300}[0.26x^{2}-0.8x+0.63];0<x<1.2\\ 0;x>1.2 \end{array}\right.$ *x*: *WSS*[*Pa*]; *R*[#/*mm*^2^]; *ρ*[#/*mm*^3^]; *t*[*sec*]	[26]
4.	Dependence of monocyte adhesion on TNF-α	$\begin{array}{l} \frac{R}{\rho t}=\frac{1}{180}\left[0.80-0.68(1-exp(-0.33{\left({log}_{10}x\right)}^{-0.56}))\right];x>1\\ \;=\frac{1}{180}[0.0996x];x\leq 1 \end{array}$ *x*: *TNFα*[*U*/*ml*]; *R*[#/*mm*^2^]; *ρ*[#/*mm*^3^]; *t*[*sec*]	[24]
5.	Dependence of monocyte adhesion on IL-1	$\frac{R}{\rho t}=\frac{1}{600}[0.14+0.80(1-exp(-0.58x^{1.19}))]$ *x*: *IL*1*β*[*U*/*ml*]; *R*[#/*mm*^2^]; *ρ*[#/*mm*^3^]; *t*[*sec*]	[27]
6.	Dependence of monocyte adhesion on WSS	$\frac{R}{\rho t}=\left\{ \begin{array}{c} \frac{1}{600}[-0.3295x^{3}+1.4x^{2}-1.8x+0.79];0<x<1.0\\ 0;x>1.0 \end{array}\right.$ *x*: *WSS*[*Pa*]; *R*[#/*mm*^2^]; *ρ*[#/*mm*^3^]; *t*[*sec*]	[28]
7.	Dependence of lymphocyte adhesion on TNF-α	$\begin{array}{l} \frac{R}{\rho t}=\frac{1}{180}\left[0.80-0.68(1-exp(-0.33{\left({log}_{10}x\right)}^{-0.56}))\right];x>1\\ \;=\frac{1}{180}[0.0996x];x\leq 1 \end{array}$ *x*: *TNFα*[*U*/*ml*]; *R*[#/*mm*^2^]; *ρ*[#/*mm*^3^]; *t*[*sec*]	[24]
8.	Dependence of lymphocyte adhesion on IL-1	$\frac{R}{\rho t}=\frac{1}{900}[0.10+0.19(1-exp(-28.29x^{1.46}))]$ *x*: *IL*1*β*[*U*/*ml*]; *R*[#/*mm*^2^]; *ρ*[#/*mm*^3^]; *t*[*sec*]	[25]
9.	Dependence of lymphocyte adhesion on WSS	$\frac{R}{\rho t}=\left\{ \begin{array}{c} \frac{1}{25}[-9.93x^{3}+11x^{2}-4.1x+0.55];0.04<x<0.41\\ 0;x>0.41 \end{array}\right.$ *x*: *WSS*[*Pa*]; *R*[#/*mm*^2^]; *ρ*[#/*mm*^3^]; *t*[*sec*]	[29, 30]
10.	Dependence of monocyte TEM on stiffness	$\%\;TEM=\left\{ \begin{array}{c} 0.78x^{2}+4.8x+51.06;1<x<5\\ 95;x>5 \end{array}\right.$ *x*: *stiffness*[*kPa*]	[23]
11.	Dependence of lymphocyte TEM on stiffness	$\%\;TEM=\left\{ \begin{array}{c} 0.78x^{2}+4.8x+51.06;1<x<5\\ 95;x>5 \end{array}\right.$ *x*: *stiffness*[*kPa*]	[23]
12.	Dependence of neutrophil TEM on stiffness	$\%\;TEM=\left\{ \begin{array}{c} 9.18x+46.96;0.42<x<5\\ 91;x>5 \end{array}\right.$ *x*: *stiffness*[*kPa*]	[31]
13.	TNF-α production	5x10^-6^ U/ml/1h/neutrophil; 6.7x10^-4^ U/ml/1h/monocyte	[32]
14.	IL-1 production	5x10^-7^ U/ml/1h/neutrophil; 5x10^-5^ U/ml/1h/monocyte	[32]
15.	IL-10 production	3.75x10^-5^ U/ml/1h/lymphocyte	[33]
16.	Dependence of directional plaque growth on plaque to lumen area ratio at each z-plane	Plaque area < 40% lumen area = plaque grows outward Plaque area ≥ 40% lumen area = 65% inward and 35% outward	[14]
17.	Dependence of wall concentration of LDL, C_w_, on WSS	$\frac{C_{w}}{t}=C_{0}(2e^{-8}x^{6}-2e^{-6}x^{5}+6e^{-5}x^{4}-1.1e^{-3}x^{3}+1.04e^{-2}x^{3}-0.05x+1.15)$ *x*: *WSS*[*Pa*]; *C*_0_ = *LDL concentration in lumen* [*ng*/μ*l*]; *t* = 1*h*	[34]
18.	LDL infiltration through wall	70% of C_w_	[34]
19.	LDL oxidation (OxLDL) rate	1.2% /1h of total LDL concentration;	[20]
20.	Monocyte to macrophage type ratio	M1(pro-inflammatory): M2(anti-inflammatory) = 2:1	[35–38]
21.	Foam cell formation	If oxLDL > 100 μg/ml, macrophage becomes foam cell	[39]
22.	Removal of IL1β by IL-10	$\frac{IL1\beta }{t}=(-0.0096x+1)\times IL1\beta$ *x*: *IL*10[*U*/*ml*]; *t* = 1*h*	[40]
23.	Removal of TNFα by IL-10	$\frac{TNF\alpha }{t}=(-0.0095x+1)\times TNF\alpha$ *x*: *IL*10[*U*/*ml*]; *t* = 1*h*	[41]
24.	Lifespan of leukocytes in wall	Neutrophils: 3 days, Monocytes: 7 days, Lymphocytes: 7 days	

# = number of leukocytes

In some cases the mechanisms to observed phenomenon such as Glagov remodeling, LDL infiltration and LDL oxidation rate (rules 16, 18 and 19) are less known. Therefore, these processes were hard-coded in the model but can be replaced by mechanistic rules as they become available.

### Main events captured in the ABM

#### Transport of cytokines and low density lipoproteins (LDLs)

At each time step cytokines, generated from leukocytes in the wall, diffuse through space (i.e. patches) in the artery wall according to Fick’s law [21]. At the lumen and outer wall, the cytokine concentration is zero due to convection (from luminal blood flow). In this model we consider two pro-inflammatory, TNF-α and IL-1β, and an anti-inflammatory cytokine, IL-10. At each time point, the total cytokine amount present in a patch is the amount of cytokine present from previous ticks plus the amount produced from all the leukocytes present in that patch plus or minus the amount diffused into it or from the patch minus the cytokines removed according to IL-10 interactions (see Table 2, rules 22 and 23).

Mass transport of LDL from lumen into the arterial wall is also an important factor for atherogenesis [42]. The majority of LDL transport occurs through leaky cell-cell junctions (> 90%) and the remainder through endocytosis [22, 43]. The occurrence of leaky junctions is governed largely by blood flow, hence WSS is considered a potent factor in LDL transport. Thus depending on shear stress, concentration of LDL in the blood, and transport kinetics, LDL enters the artery wall (see Table 2, rules 17 and 18). LDL then diffuses spatiotemporally according to Fick’s law. LDL is oxidized in the arterial wall and this oxidized form of LDL (ox-LDL) is consumed by monocyte derived macrophages forming foam cell (Table 2, rules 19 and 21) [44].

#### Adhesion and leukocyte TEM

Leukocyte adhesion and TEM is a two-step process. First the probability of leukocyte adhesion (p) to the endothelium depends on endothelial cell activation via cytokine concentration, on WSS and on concentration of leukocytes present in the blood. In our ABM, the rules of adhesion are of the form:

$$\frac{R_{i}}{\rho_{i}t}=\frac{f_{i}(\alpha )}{t_{exp}}$$ (1)

Where index *i* represents lymphocyte, monocyte or neutrophil, R_i_/t is number of leukocytes of type *i* that adhere per unit surface area (leukocytes/mm^2^) per time, ⍴_*i*_ is the density of the leukocytes of type *i* in the blood (leukocytes/mm^3^), t_exp_ is time duration of the experiment and *f(α)* are experimentally derived functions, where *α* represents WSS, TNF-α and IL-1β (see Table 2, rules 1–9), specific to each type of leukocyte. f(α) is derived from the published literature by first plotting R_i_ /⍴_*i*_ versus α and using matlab regression algorithms to determine the best fit equation to resent the data. It has been found that best fit function for leukocyte adhesion based on endothelial activation by cytokines (TNF, IL-1) are sigmoid functions (rule 1,2,4,5,7,8), whereas the best fit functions for leukocyte adhesion dependent on WSS (rule 3,6,9) are polynomial of order two or more.

In the model, the probability of adhesion (p) can be computed for each leukocyte type. Fig 1 illustrates a luminal and adjacent artery patch containing leukocytes and ECs, respectively. N is the total number of leukocytes present in the luminal patch of volume, V (mm^3^). Among N, M is number of leukocytes that will adhere to the endothelium of surface area, A (mm^2^). Hence according to law of large numbers (LLN), the probability of adherence (p) is M/ N. Since, volume V = Ah, where h is the patch length (mm), the following relation can be derived to compute the probability of leukocyte adhesion by type, p_i_.

$$\frac{R_{i}}{\rho_{i}t}=\frac{M_{i}/A}{N_{i}/V}\frac{1}{t}=\left(\frac{M_{i}}{N_{i}}\right)\left(\frac{V}{A}\right)\frac{1}{t}=\frac{p_{i}h}{t}$$

$$∴p_{i}=\frac{f_{i}(\alpha )}{h}\frac{t}{t_{exp}}$$ (2)

Second, leukocytes transmigrate through the endothelium depending on the arterial stiffness. TEM increases with arterial stiffness (see Table 2, rules 10–12).

#### Leukocyte chemotaxis

**Fig 2:**
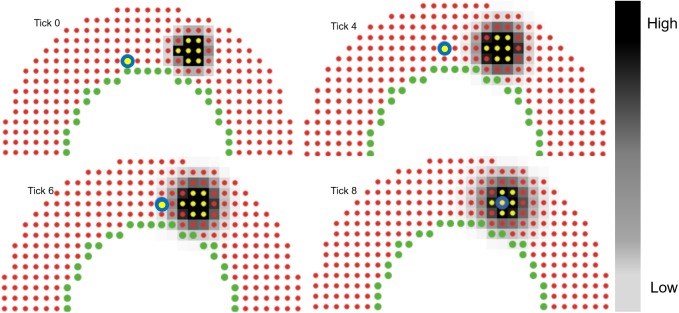
Protein diffusion and leukocyte chemotaxis. 2D example of leukocyte (yellow circle with blue outline) migration. Color bar represents the cytokine concentration gradient (black (highest) to white (lowest) via gray). Yellow agents produce the cytokines. At tick 0 the leukocyte is 5 patches away from the source. Cytokines diffuse at each tick following Fick’s law. At tick 4, the leukocyte moves to its topmost neighboring patch, since that patch has the highest cytokine concentration and space available. In next ticks, the leukocyte surveys it’s neighboring patches and moves to the one with space available and the highest concentration. At tick 8, it has reached the patch with the highest concentration (darkest). Each tick represents 1 hr.

Upon entering the artery wall, leukocyte migration is guided by chemotaxis, i.e., directed cell movement towards higher concentrations of chemoattractant. Since our goal is to model leukocyte TEM and plaque evolution as a function of hemodynamics the wall composition is considered uniform throughout, with no distinction between media and adventitia. 60% of the arterial wall is considered fixed with ECM, namely collagen and elastin, and the remaining 40% is composed of cells and soluble factors [45–47]. Specifically, 40% of each patch of the wall is available for leukocytes, macrophages and foam cells. Once the leukocytes adhere to the wall they first determine which neighboring patch has space available and second, of those with available space which one has the highest concentration of pro-inflammatory cytokines and finally moves towards that patch based on its migration speed. This process repeats, within a tick and at each following tick, until the leukocyte finds the patch with space and the highest concentration of cytokines as compared to neighboring patches. Fig 2 illustrates the chemotactic process using a 2D ABM. To save computational time, in the case of multiple leukocytes undergoing TEM at the same patch at the same time, each leukocyte will follow the same chemotactic path during that tick (if the patch is not completely filled). This save computational time since after finding the path for one leukocyte, all the remaining leukocytes from that patch go straight to the final patch as found by the first leukocyte.

#### Glagov remodeling

According to Glagov remodeling, [14] as the plaque area increases inside the artery wall the lumen area remains preserved until the plaque area is 40% of the lumen area at that specific axial-plane. After which the plaque starts growing inward thus reducing the lumen area. To determine this point, at each time tick in the ABM, the plaque area along the axial length (in segmented intervals of one patch) of the artery is compared to the lumen area of the corresponding segment. If the plaque area is greater than 40% of the lumen area then that axial segment of the plaque starts growing inside the lumen in addition to outward growth (see Table 2, rule 16). Plaque area is considered as number of patches with macrophages, leukocytes and foam cells whereas lumen area is considered as number of patches in the lumen of the corresponding axial plane.

#### Rule confidence

Since the model is only as good as its rules, and each rule is derived from different sources we used a rule scoring metric to gain confidence in each rule, as previously described [48]. Briefly, each rule is independently evaluated for 1) universal acceptance by different published literature sources, 2) physiological accuracy of the articles methods, 3) similarity of the conditions in the article to those being simulated in our ABM, and 4) precision of the data measurements. See S1 Table for all the scores from three blinded researchers. If the average score for a given rule is under 5 it is deemed less reliable. Thus we either rederived the rule (e.g., formulating the rule from one of the articles making a contradictory but more prevalent claim) or generalized the correlation if limited data exists.

#### Computational fluid dynamics (CFD)

As the plaque grows inward (according to Glagov phenomenon) the change in luminal geometry alters the blood flow. Therefore, CFD analysis is used to investigate the hemodynamic effect of the plaque burden in the left coronary artery. This investigation gives spatiotemporal information about WSS, which in turn is used to predict the leukocyte transmigration. A commercial CFD package COMSOL (5.2a) is used for this simulation.

#### Conditions of the CFD model.

**Fig 3:**
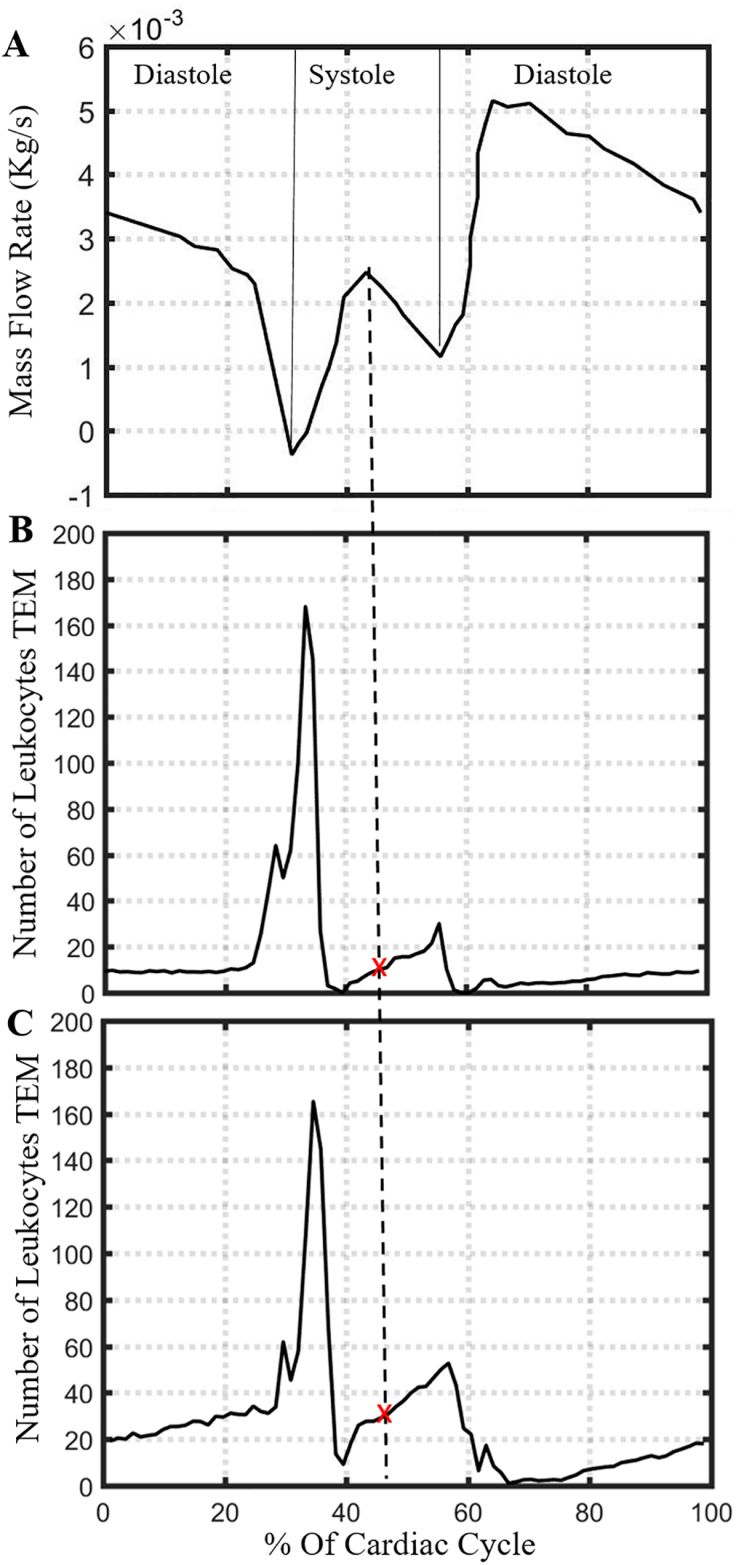
Representative cardiac cycle to pass spatial WSS profile into ABM. (A) Coronary blood flow rate profile used in CFD. Number of leukocytes undergoing TEM in 1 hour using instantaneous pulsatile flow over a spherical plaque with radius (B) 1.0 mm or (C) 1.5 mm. The average number leukocyte TEM per hour, over one cardiac cycle, is 16 for (B) and 26 for (C). The WSS profile at peak flow during systole corresponds to these average TEM values, as indicated by the dashed line and red ‘x’ marks.

- *Geometry*: The luminal surface of the artery, according to ABM, is passed to COMSOL. Specifically, a text file containing the position of each EC, at the centroid of cubical patches, is first passed to MATLAB (R2016a). A MATLAB routine then reconstructs the inner layer by sorting all the x and y points in the azimuthal direction for each z-plane and smooths the shape by first taking the average of two consecutive x, y points in the azimuthal direction and then applying the built-in smooth function in MATLAB (S1 Fig), and finally creates a surface mesh stereolithographic (STL) file. The STL file is then imported in COMSOL. The wall is considered rigid over the WSS calculations (see discussion section).
- *Blood*: Density and dynamic viscosity of blood are 1060 kg/m^3^ and 3.5e-3 Pa-s respectively. Blood is considered homogeneous, incompressible and Newtonian.
- *Flow and boundary conditions*: Laminar pulsatile blood flow (Fig 3A) with a cardiac period of 0.8 sec is introduced at inlet where the wall is considered rigid and cylindrical [49]. Nosovitsky et al. validated the assumption of laminar flow in stenotic arteries is appropriate [50]. No slip at wall and constant pressure at the outlet are used.

#### Determination of the optimal timescale to pass WSS into ABM

Since the blood flow is pulsatile in nature, the WSS and rate of leukocyte TEM changes throughout the cardiac cycle. Utilizing 3D WSS values at each instant in the cardiac cycle is not practical due to the time scale of atherogenesis, which occurs over the span of years. Uniform or constant WSS values corresponding to average flow rate would not be physiological since they would not capture the low end of WSS values which are, according to rule, chiefly responsible for TEM.

To determine the appropriate point of the cardiac cycle to calculate WSS from, WSS is computed using pulsatile flow over two spherical plaques (one of radius 1.0 mm and other 1.5 mm) and WSS saved at each 0.01 sec interval for each lumen patch location. The average number of leukocyte TEM over one full cardiac cycle can then be determined and a representative time point identified. This value is compared to steady flow and used to determine the appropriate flow rate that will generate approximately the same total leukocyte transmigration over a cardiac cycle. Future simulations pass the WSS calculated at this time point within the cardiac cycle.

#### Development of a comprehensive leukocyte TEM model

**Fig 4:**
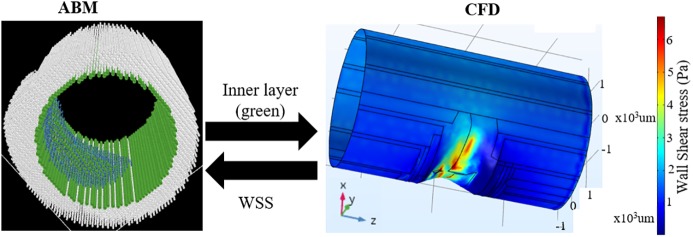
Multiphysics model showing the handshaking between the ABM and CFD. The coordinates of the inner arterial layer from ABM (left) is sent to COMSOL to perform CFD analysis (right). The instantaneous WSS from CFD is sent back to ABM and influences leukocyte TEM.

As mentioned in the introduction, our goal is to develop a comprehensive model of leukocyte TEM. This model includes the effects of biological (ABM) and hemodynamic (CFD) factors to predict leukocyte transmigration and G&R of a LAD in atherogenesis. When the plaque grows inside the lumen, the hemodynamics is altered. Therefore, given an ABM geometry, CFD is performed to determine the WSS, which is then sent back to ABM to simulate leukocyte transmigration (Fig 4).

#### Determining rate of leukocyte TEM

To initially determine how many leukocytes enter the artery wall as a function of WSS and plaque shape we have used an accelerated G&R version of ABM whereby only 4%, instead of 40%, of the patch is available for leukocytes. Specifically, after a change in internal geometry, i.e. change in number of luminal patches, the ABM was paused, CFD is performed with the current geometry, and WSS was updated into ABM and ABM restarted. This method was used starting with three spherical plaques of different severity, small (0–5% stenosis), medium (5–20% stenosis) and big (20–35% stenosis) (S2 Fig) and the leukocyte transmigration was found as a function of only WSS. Using these accelerated models we found leukocyte TEM over a given change in luminal volume. This constant zone decreased with plaque size. To confirm the rate of leukocyte TEM, found using spherical plaques, we updated the WSS at each change in luminal patch in a non-accelerated model where TEM is a function of WSS as well as endothelial activation.

### Confidence in the model

#### Verifying model stability

To gain confidence in model, we induced a temporary spike (at each 10 hour) in cytokine concentration or WSS and ensured homeostasis (i.e. balanced production and removal) over one week (S3 Fig). The artery does not chronically remodel to these temporary blips.

#### Verifying spatial WSS distribution and leukocyte TEM

Leukocyte TEM is inversely proportional with WSS. Therefore, we plotted WSS (from CFD) with the spatial positions of leukocyte TEM at the lowest flow rate (33% of cardiac cycle) and the highest flow rate (6% of cardiac cycle). As expected, transmigration was highest and distributed over the plaque evenly under low flow conditions. Conversely, the level of transmigration was low and WSS high under higher flow conditions. S4 Fig illustrates the WSS distribution over a stenosis with overlaid transmigration positions, as obtained from the ABM (red dots).

## Results

### Capturing instantaneous WSS effects in the ABM

We identified a simplifying assumption for handshaking between the ABM and CFD that generated approximately the same total leukocyte transmigration as if we include the complete pulsatile flow history. Fortunately, over an entire cardiac cycle, the average number of leukocytes undergoing TEM per hour, updating the WSS distributions at each 0.01 second of the pulsatile flow increment, for an artery with a spherical plaque of radius of 1mm or 1.5mm, are 16 and 26 respectively (Fig 3B and 3C), which were recovered using only the WSS distribution at the peak coronary flow during systole, i.e., between the opening and closing of the aortic valve. In contrast, with an input of mean steady flow, an average of only 7 leukocytes transmigrated into the wall after 1 hour. Thus a steady flow profile was inadequate but a judicious choice from the pulsatile flow case can provide a computationally tractable alternative.

### Leukocyte TEM increases with the level of stenosis

**Fig 5:**
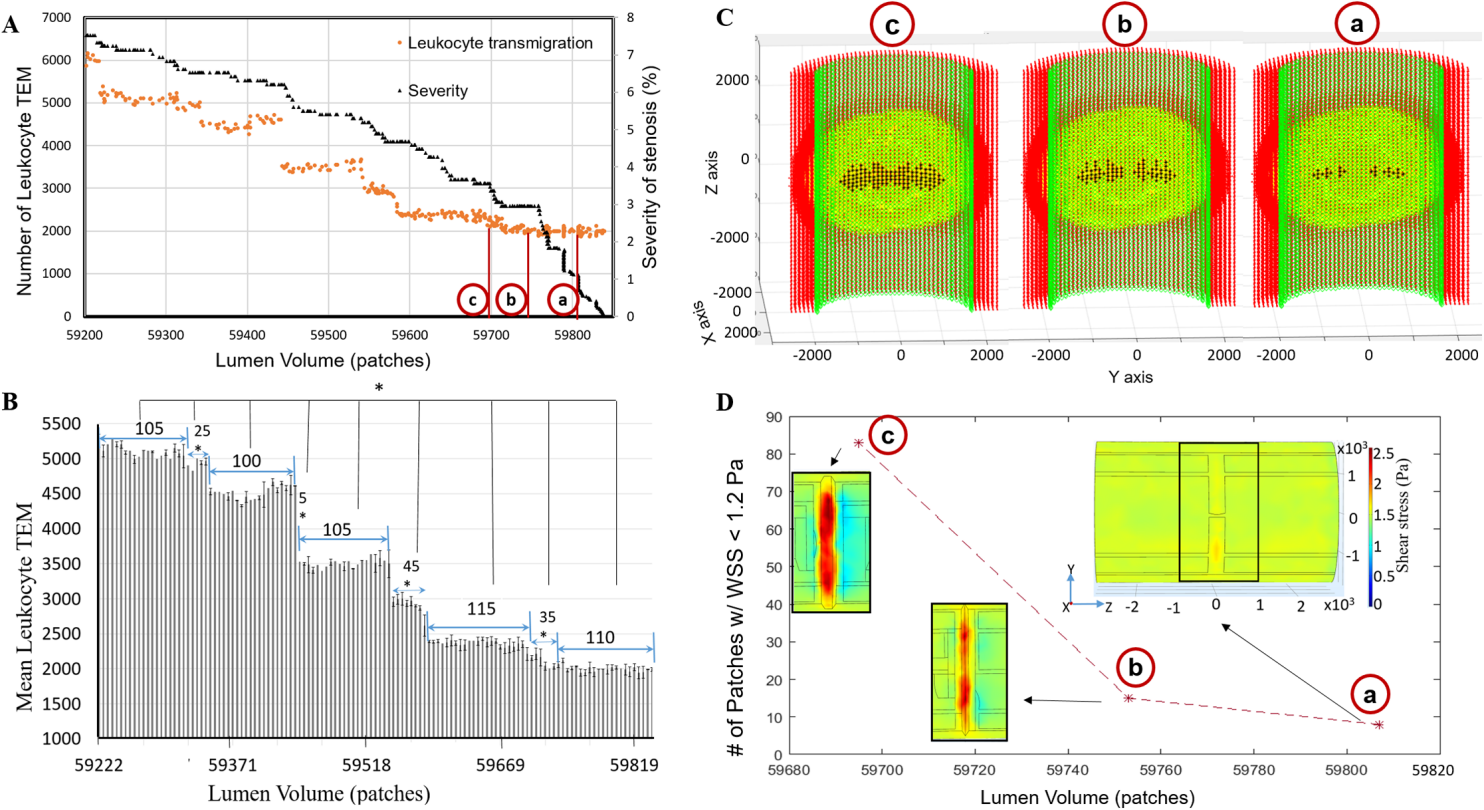
When the degree of stenosis was below 8%, the level of leukocyte TEM was constant over an average change in lumen volume of 107±5 patches, followed by a rapid increase in TEM over the next 28±17 patches. (A) Scatter plot displaying leukocyte TEM and severity of stenosis as a function of luminal volume (patches). (B) Bar plot indicating the change in lumen volume after which a significant increase in leukocyte TEM was observed. (Mean ± SD, *p < 0.05). (C) Longitudinal cross sections of the ABM at ‘a’, ‘b’, and ‘c’ points from 5A, illustrating where the initial inward growth occurred. ACs, ECs, and leukocytes in the plaque are indicated in red, green and yellow, respectively. Black represents the new ECs added in the lumen. Inlet flow is at the bottom of each subfigure. (D) Subplot showing the number of patches (from ABM) with WSS < 1.2 Pa at specific plaque shapes (i.e., lumen volumes) corresponding to ‘a’, ‘b’, and ‘c’ points from 5A. Overlaid color contour plots of the WSS (from CFD) over the plaque at each point. The number of patches of low WSS (blue region) are almost constant (13 and 15 respectively) corresponding to nearly constant TEM. Then, the number of patches having low WSS increases (83) as does TEM. Inlet flow is at the left of each subfigure in (D).

Leukocyte TEM increased monotonically as the plaque grew into the lumen over time and so does severity (Fig 5A). Initially, we created spherical plaques, varying in severity of stenosis, into an accelerated version of the ABM and monitored leukocyte TEM and plaque growth. Interestingly, we observed the rate of leukocyte TEM was not constant as the plaque grows, that only a few leukocytes transmigrated followed by a significant increase in leukocyte TEM. These increases occurred after luminal changes of 80, 35 and 10 patches (lumen volume corresponds to number of patches in the lumen) when the severity of stenosis was between 0–5%, 5–20%, and 20–35%, respectively (S2 Fig). When plaque severity was below 8%, our non-accelerated ABM predicted significant increases in leukocyte TEM after growing by 100 to 115 (average ± standard deviation 107 ± 5, n = 5) patches into the lumen (Fig 5, p < 0.05). Together, these results corroborated that during initial plaque growth (i.e., level of stenosis < 8%) the plaque will grow inside of the lumen by at least 80 patches or 0.08 mm^3^ before a significant increase in leukocyte TEM was observed. Fig 5B illustrates between two constant zones there was a “transition zone” of about 28 patches where the rate of TEM increases significantly. Next we used the ABM to better understand what spatiotemporal conditions lead to these transition zones.

figVariations in WSS, as compared to cytokine concentrations, have a greater influence on leukocyte TEM. Fig 5C and 5D highlight two representative plaques undergoing similar levels of leukocyte transmigration (points a and b) and one representative plaque experiencing significantly higher rates of leukocyte TEM (point c). In these three plaques, the endothelial patches with the highest cytokine concentration (1.6 ± 0.06 ng/ml TNF, 0.18 ± 0.01 ng/ml IL-1) were near the minimal luminal area (MLA) or maximum stenosis. WSS was also the highest (> 2 Pa) at the maximum stenosis and lowest (< 1.2 Pa) in the distal and proximal zones. However, leukocyte adhesion due to low WSS was almost 10 times higher than due to the highest cytokine concentration. Hence most of the leukocyte TEM occurred in the distal zone of the plaque where the WSS was favorable for TEM (i.e. < 1.2 Pa, 5D). For example, when the lumen volume was 59807, 59753 and 59695 the number of endothelial patches with favorable low WSS were 8, 15 and 83 respectively. Hence leukocyte TEM was nearly constant with a luminal geometry of the first two points, after which TEM increased significantly (transition zone) (Fig 5D). More leukocytes entering the artery wall caused the plaque to grow or expand inwardly. Fig 5C illustrates the locations where the plaque start to protrude into the lumen during the constant zone (points a and b) and at the higher TEM zone (point c).

### Model of atherogenesis reveals spatiotemporal G&R

**Fig 6:**
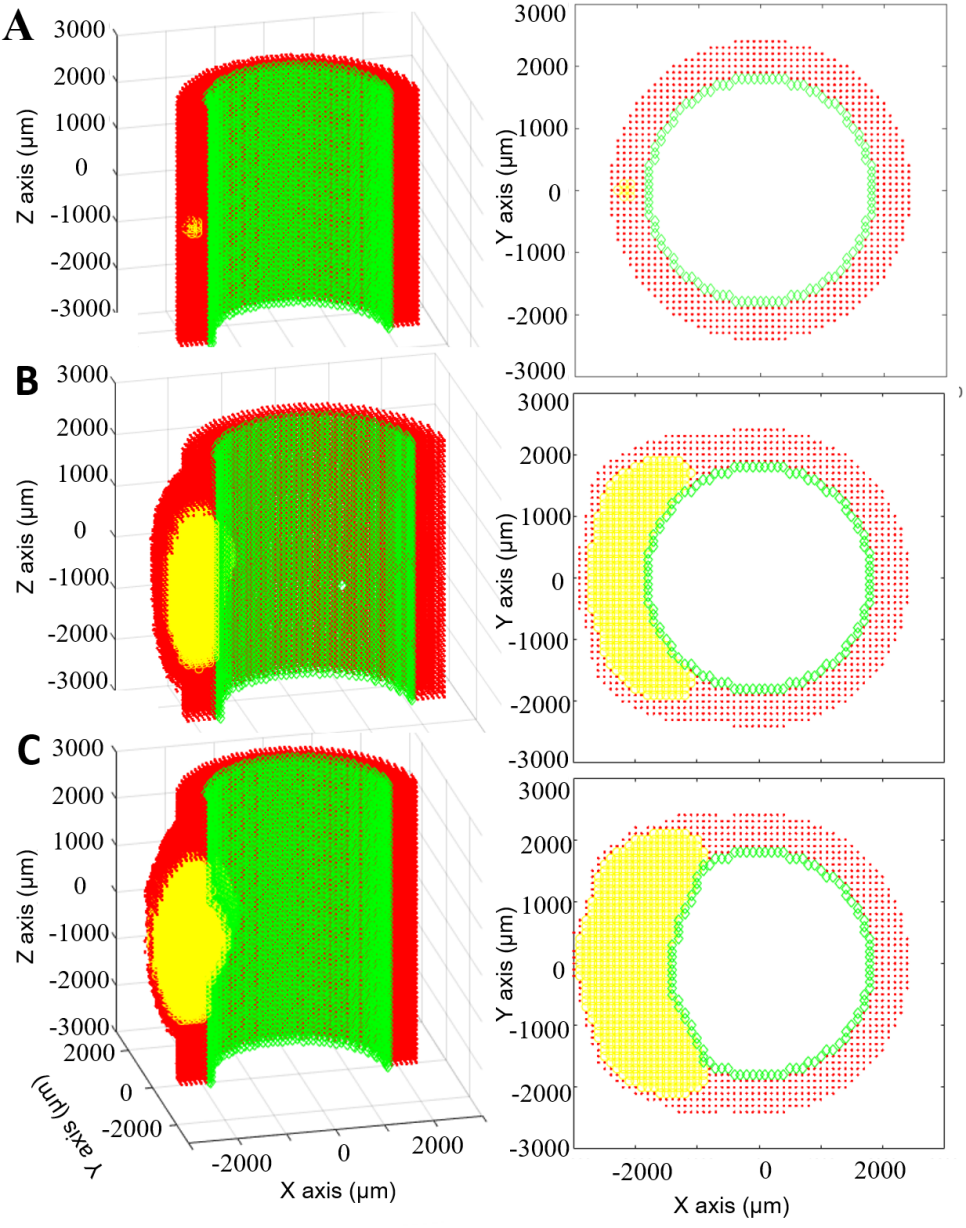
Eccentric plaque growth and remodeling prediction. Longitudinal (left) and corresponding transverse (right) views of an evolving artery where ECs, ACs and leukocytes are represented by green, red and yellow respectively. A) Initially the artery is impregnated with 15 leukocytes. B) At 6 months the plaque area is 40% of the lumen area and will start growing inside lumen according to Glagov’s phenomenon. C) At 7 months the plaque has grown inward and outward, changing the luminal geometry.

**Fig 7:**
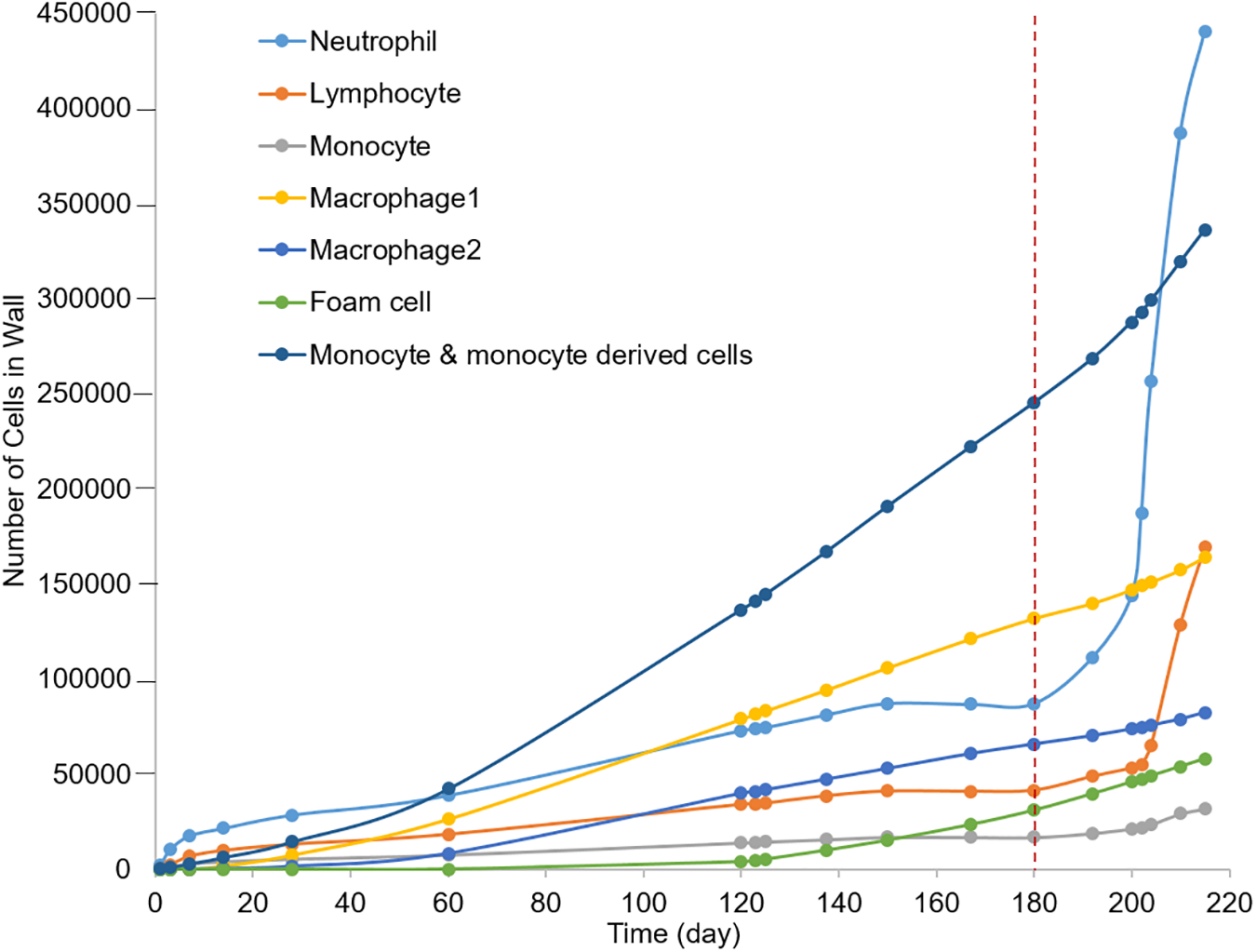
Different types of leukocytes present in the plaque as it evolves. Before the plaque reduces the caliber of the lumen, as indicated by the vertical dashed red line, TEM is only due to endothelial activation by cytokines. Initially neutrophils constitute the majority of the plaque volume, followed by monocytes and monocyte-derived cells and then neutrophils again. When the plaque starts growing inside the lumen (vertical dashed line) leukocyte TEM is largely influenced by blood flow. With time the severity of stenosis increases and so does the region of low WSS. Therefore, the rate of leukocyte TEM is greater for all cells. Among these cells, the concentration of neutrophils (62%) in blood is higher than monocytes and lymphocytes (5.3% and 30% respectively). Also neutrophils adhere on the EC surface with WSS < 1.2 Pa whereas the monocytes and lymphocytes adhere with WSS < 1 Pa and < 0.4 Pa respectively. Thus there is a rapid increase of neutrophils immediately after 6 months whereas the rate for lymphocyte increases after several days when the plaque is bigger and WSS < 0.4 Pa.

Fig 6 illustrates the ABMs ability to capture growth and remodeling of the LAD coronary after an initial insult of 15 leukocytes placed in the middle of the arterial wall (Fig 6A). The cytokines generated by these leukocytes activated the local endothelium leading to leukocyte adherence and TEM. Once in the wall, the leukocytes migrated, differentiated, undergo apoptosis, phagocytosis and synthesize proteins based their behavior rules and environmental conditions. The sum of these events resulted in outward arterial remodeling until the plaque area was 40% of lumen area (Fig 6B). As expected acutely neutrophils were the predominate leukocyte cell type in the wall (Fig 7). After 60 days of growth monocytes and monocyte-derived cells became the majority. Once the plaque started growing predominately inward, at select z-planes (Fig 6B), altered blood flow gave rise to lower WSS in the distal regions as compared to the maximal lumen stenosis (S4 Fig). Interestingly, the ratio of neutrophils within the plaque began to increase again (Fig 7). After about a month of inward growth the ratio of lymphocytes within the artery increased. Together the increased leukocyte adhesion resulted in accelerated plaque growth once it started to stenosis the lumen volume (Fig 6C).

### Model sensitivity and stochasticity

Neutrophil adhesion is more dependent on IL-1β than TNF-α. According to Bahra et al. [24] and Breviario et al. [25] when the concentrations of IL-1β and TNF-α are low (< 1 U/mL), neutrophil adhesions is primarily due to IL-1β, whereas if the concentrations are high (> 1 U/mL) the reverse is true (S5B Fig). From the model we found that the highest concentration of either IL-1β or TNF-α in an endothelial patch over a 50 day simulation is below 0.04 U/mL (S5A Fig). That is the probability of neutrophil adhesion due to the maximal concentration of IL-1β in an endothelial patch is 0.247, whereas the probability of adhesion due to the maximal concentration of TNF-α is only 0.003. Indeed, even after 7 months the IL-1β is 0.006 U/ml and TNF- α concentration is 0.07 U/ml, meaning the probability of adhesion is still influenced more by IL-1β than TNF-α (0.253 and 0.008, respectively). Similarly, the probability of adhesion of monocytes and lymphocytes (according to rules 4, 5, 7, 8) is greater for the predicted endothelial concentrations of IL-1β than for the predicted endothelial concentrations of TNF- α.

Prior to stenosis, the model exhibits little run-to-run variation. To determine the stochasticity of the model, we repeated the simulation three times. Supplemental S6 Fig shows average number (from three simulations) of different cell types in the wall and the shaded region shows the standard deviations.

### Tissue-level validation of the model with an experimental study

**Fig 8:**
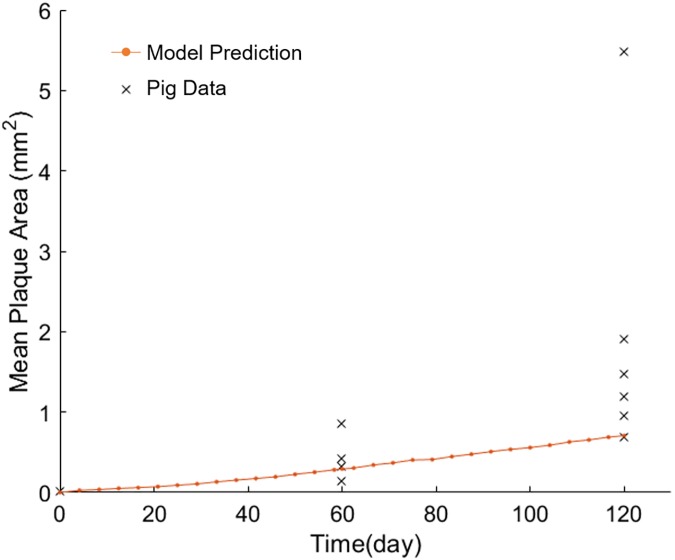
ABM-CFD predictions corroborate an experimental porcine model of atherogenesis. ‘x’ represents mean plaque area from individual pigs, fed high cholesterol diet. ‘.‘represents predicted plaque area as a result of a 30% increase in LDLs.

Predicted plaque area with time was compared to that observed in a pig model of atherogenesis. The coronary hemodynamics and atherosclerotic lesion morphology in the pig heart closely resembles those seen in the human heart [51–53]. Pelosi et al. [54] studied atherogenesis in the coronary of pigs at 0, 2, and 4 months of high cholesterol diet. They measured mean lesion area of 4–5 cross sections, each separated by 0.5 mm. The ABM initially mimicked the conditions of the experimental pig model in regards to wall composition, blood hematocrit and 30% increase in LDL at day 0. The model simulated 4 months, and the mean lesion area was determined using 5 cross sections, 0.5 mm apart and centered about the middle of the plaque. The results (Fig 8) were similar to the plaque growth seen in the pigs. Thus, simulated results corroborated the experimental porcine model at the tissue-level.

## Discussion

Herein we present a novel way to predict leukocyte TEM and overall G&R of a LAD coronary artery during atherogenesis. We developed an ABM to capture the spatiotemporal effects of hemodynamics on leukocyte adhesion, transmigration and plaque formation. Previously, a 2D ABM was employed to study vascular remodeling due to a focal stenosis (via ligation) in a rabbit vein graft model [55]. In the study, the exerimental model and their 2D ABM predicted significant intimal thickening, via mostly smooth muscle cell proliferation and extracellular matrix production, in the low WSS zone distal to the stenosis. Likewise, our 3D ABM predicted increased wall thickening in low WSS zones. Yet the increased thickness was due to an increase in extremal mass (i.e. leukocyte infiltration) rather than cell proliferation. Moreover, we present an ABM-CFD modeling approach that accounts for instantaneous fluctuations in WSS throughout the cardiac cycle as well as updates the WSS after significant changes in geometry, as opposed to updating the WSS at an arbitrary frequency. In the next generation of the presented ABM-CFD model we plan to include the rules related to residual artery wall cell and ECM to tease out the influence they have on plaque development. Recently, Olivares et al. used ABM to parametrically identify oxLDL, out of other parameters such as cell migration, statins and auto-antibodies, as the most influential factor in macrophage to foam cell transition during early atherosclerosis [56]. However, being chiefly a parametric study, they did not include important interconnected events such as hemodynamic regulation of LDL and leukocyte TEM, chemotactic-guided cell migration, cytokine synthesis by the various leukocytes that invade the artery wall (i.e., lymphocyte, monocyte, leukocyte, macrophage (M1, M2) and foam cell), or cytokine induced endothelial activation. The ABM presented herein introduces an original way to capture these complex interconnected events. ABM is a promising tool to understand how spatiotemporal changes influence plaque progression and how complex and integrated processes give rise to emergent phenomenon.

Our mechanobiolical ABM builds on, and is in agreement with, previous multi-level modeling attempts to capture hemodynamically inspired inflammation and atherogenesis. In 2007, Bailey et al. used a 2D ABM of mouse to capture WSS- and chemokine/cytokine-induced monocyte adhesion to ECs of microvessels [57]. Similar to our results they found monocyte adhesion to the endothelium increases under low WSS and decreases once the WSS crosses the maximum threshold to withstand leukocyte adhesion. In 2012, Filipovic et al. used finite element modeling and a series of reaction-diffusion equations to look at LDL transport into the lumen and growth of an advanced stenosis. Recently in 2016, this group expanded on these studies by developing a multi-level numerical model capable of capturing WSS-induced monocyte TEM and predicting areas particularly susceptible to plaque growth [58]. Similarly, we also report a positive correlation between low WSS regions and LDL accumulation and plaque growth. The model developed herein is unique and novel in comparison to the above mentioned models in that it includes multiple types of leukocytes, not just monocytes, it has continuous and/or stochastic rules rather than discrete and deterministic rules, cell migration is based on chemotaxis rather than convection-diffusion, and it allows the plaque to evolve non-symmetrically while observing tissue-level Glagov phenomenon rather than axisymmetric steady state simulations. Collectively these added features predict monotonic plaque growth with periodic increases in leukocyte TEM.

As expected, leukocytes entered the arterial wall and migrated towards the center of the plaque according to chemotaxis. When the plaque was > 40% of the lumen area it started growing inward (i.e. towards the lumen). Interestingly, new luminal patches were first added near the center of the plaque but laterally from the centerline (see Fig 5C, (a)). We used the model to determine the mechanisms behind this phenomenon. First, we observed that most of the endothelium near plaque constituents is activated; second, and leukocytes entering the wall from the edges or “shoulder” regions of the plaque migrate to the center. Third, because the plaque has advanced within the artery wall, the first location the leukocytes find with space still available and the highest cytokine is just off the centerline. Therefore, these lateral regions exceed 40% of the luminal area before the centerline does and are the first to protrude into the luminal space. Once several luminal patches have been added as to form a layer over these lateral regions, leukocyte TEM occurs at a more rapid rate (Fig 5C, (c)). At this point in time, inward growth starts occurring in the centerline of the artery resulting in more patches with WSS < 1.2 Pa (blue regions in Fig 5D). As a result, the model predicts a “transition zone” where an increase in the rate of leukocyte TEM is observed. After ~28 luminal patches are added, the number of leukocytes performing TEM levels as luminal patches are mostly added to the lateral regions again. These results corroborate with the general idea that the height of the stenosis appreciably influences the low WSS zones [59]. Herein we use our ABM to provide insight as to how the integration of hemodynamics, TEM, chemotaxis, and remodeling phenomenon influence plaque shapes.

Clinically, hemodynamics has been used to predict areas of plaque progression [10, 60]. Stone et al. used coronary angiography and intravascular ultrasound (IVUS) to reconstruct the artery and calculate WSS from 374 CAD patients (2.7 coronary arteries per patient) at baseline and 6–10 months later [60]. They found that regions with low WSS (< 1 Pa) distal to the throat of the obstruction grew most rapidly (with almost 30% of the patients experiencing a decrease of luminal area > 2.4 mm^2^). Samady, et al. went one step further and correlated local WSS with plaque composition using IVUS-virtual histology (IVUS-VH) in 20 patients with CAD over 6 months [10]. They also found arterial segments with low WSS (< 1 Pa) developed greater plaque progression (0.12 ± 7.8 mm^2^) and reduction in lumen area (by -0.9 ± 1.5 mm^2^) as compared to higher WSS areas. Moreover low WSS regions experience an increase in fibrofatty area (0.01 ± 0.09 mm^2^) compared to the high (> 5 Pa) WSS regions (-0.14 ± 0.44 mm^2^) which actually experience regression of fibrofatty tissue. Smedby performed angiography on 237 patients over a span of 3 years to find that plaque growth was more rapid downstream of the stenosis, as compared to upstream [61]. In agreement with these clinical observations, our model also shows a positive correlation between low WSS zones (< 1.2 Pa) and plaque progression. Fig 4 illustrates the low WSS region in the downstream, or distal, shoulder region of the stenosis. We observed increase leukocyte TEM in this region (S4 Fig) followed by local plaque growth. In addition, as a form of gross validation we simulated the same conditions as an experimental porcine model of atherogenesis. Our ABM-CFD mean lesion area predictions were similar to those quantified in the experimental model (Fig 8) at 2 and 4 months. However, at 4 months, the lesion area as predicted by the ABM-CFD model was in the lower range. This may be due, in part, to the fact that the plaque is comprised exclusively of leukocytes and ECM in the ABM, whereas in reality there may be migration and proliferation of smooth muscle cells and fibroblasts as well as production of additional ECM all of which could increase the lesion area. Yet, the ABM-CFD lesion area predictions highlight the influence of leukocyte accumulation on plaque development.

At the cellular-level, the ABMs spatial and temporal predictions on ratio of cell types within the artery wall corroborate with experimental and histological reports. We found the relative amount of neutrophils was the highest in the beginning (within ~ 2 months) and then again about a month after when the plaque started to grow inside the lumen (Fig 7). Mechanistically, neutrophil aggregation can be explained by the higher concentration of neutrophils in the blood and their increased adhesion rates under lower cytokine and higher WSS conditions (Table 2, rules 1–3). A review by Soehnlein eloquently highlighted animal studies showing significant neutrophil involvement during the initial progression of atherosclerosis as well as during endothelial dysfunction as may be the case when the endothelium is disrupted by inward plaque growth [62]. Indeed the rapid influx of neutrophils and then lymphocytes after inward remodeling may explain the occurrence of plaque fissures in minimally stenotic plaques [3]. Spatially, by 7-months the model predicted neutrophils and monocytes coalesce towards the adventitia, cap, and shoulder regions of the plaque (S7 Fig). Similarly, Rotzius et al. showed in mature lesions, neutrophils were abundant in the shoulder region of lesions, especially at the sites where the concentration of monocytes were also high. The ABM-CFD model can also be used to identify the global affect due to alteration of specific factors. For example, Stoneman et al. [63] depleted monocytes in ApoE knockout mice and showed a 50% reduction of plaque area after 10 weeks. Removing monocytes from the ABM resulted in around 80% reduction in plaque area by 10 weeks. As expected, both models showed a reduction in plaque growth; the difference in magnitude may be due to genetic effects of the ApoE knockout, proliferation of the arterial cells and/or production of ECM.

### Conclusion

In conclusion, by considering key biological events such as diffusion of cytokines and LDL, cytokine- and WSS-stimulated leukocyte adhesion and trans-endothelial migration, chemotaxis, cell apoptosis, monocyte differentiation, and foam cell formation, our ABM can capture and predict leukocyte TEM and thus plaque progression. Moreover coupling this ABM to a blood flow model allows for a better understanding of the spatiotemporal hemodynamic effects on plaque progression. Experimentally, the dependency of TEM on endothelial activation and WSS has been quantified [23–31, 64, 65]. Clinically low WSS has been linked to areas of plaque growth and luminal constriction [66, 67]. Now, computationally we present a robust multiscale model to study the interdependency of leukocyte TEM, plaque growth and WSS with time. Together, it provides an accurate approach to predict atherosclerotic plaque dynamics and avoids the homogeneous idealizations or isolated correlations of existing approaches. Eventually, multi-scale modeling of plaque evolution will be insightful for individualized decision making (e.g. to treat or not to treat a lesion) and foundational for design changes in interventional approaches (e.g. hypothesizing how an artery will respond to a pharmaceutical candidate, stent design or graft).

### Assumptions and limitations

Longitudinal human data during atherogenesis, where the severity of stenosis is < 10%, does not exist. We cannot directly compare our growth rate predictions, though the model’s growth rate (i.e., going from 0 to 8% stenosis in 6 months) seems accelerated. One reason may be due to fact that the rules on leukocyte adhesion and TEM are based on experiments conducted in-vitro. However, we have tried to minimize error within each rule by having them tested for robustness by blind researchers. Another reason may be spatial restrictions in the artery wall. As the primary focus was on spatiotemporal WSS-induced leukocyte transmigration we have fixed the ECM as 60% of each patch in the artery wall. In reality, matrix metalloproteinases, released by cells degrade the ECM and thus free up more space and slow the growth rate. Even so, the growth rate should not affect the dynamics observed on how plaque stenosis alters the hemodynamics and thus leukocyte transmigration. Moreover, comparing the model predictions on lesion area were similar to a pig model of atherogenesis.

As previously mentioned, each patch was a cube of length 100μm. Hence all the leukocytes present in same patch at a time experienced the same environment (i.e., cytokine concentration and WSS). Obviously smaller patch sizes would increase the resolution, but also the computational time. Spatial results confirmed that the cytokine concentration in one patch did not change much from the next patch even in the most concentrated zone (S8 Fig). Likewise, Razavi et al. showed the WSS changes by less than 2% within 100 μm, even in the case of 60% stenosis [68]. For a reasonable computational time as well as capturing the variations of the parameters properly, the patch size was considered as 100μm.

A rigid wall assumption was imposed. Differences of WSS between compliant and rigid wall models depend on several factors (e.g., degree of compliance, geometry, curvature, and stenosis severity). Coronary arteries with mild stenosis, exhibit a change in diameter < 2% over a cardiac cycle and peak WSS differences between compliant and rigid wall models is < 10% [69]. In arteries with severe stenosis the difference is around 30–40% [70]. Therefore, a rigid wall assumption was a close approximation for the case of early atherosclerosis studied herein.

## Supporting information

S1 Table Rule scoring results. (XLSX)

S1 Fig Inner layer data from ABM: Left: Cross section of the lumen where plaque is already inside lumen. Since agents in ABM are at centroid of patches, the inner layer is a saw-toothed line. Middle: Smooth surface of the inner layer. Right: STL file generated in MATLAB.
(TIF)

S2 Fig Frequency of updating WSS: WSS is updated at each change of geometry (lumen patch) and corresponding leukocyte TEM is obtained from ABM. It is found that for small (A), medium (B) and big (C) spherical plaques, up to change of lumen patches of 80, 35, 10, respectively. TEM is almost constant. Hence WSS update is necessary after change of 80, 35, and 120, respectively.
(TIF)

S3 Fig **ABM ensures homeostatic and stability under temporary spike:** (A) the model is run with normal WSS (1.4 Pa) and inactivated endothelium. As expected no transmigration (left) as well as no plaque growth (right) is overserved. (B) An example result of the model under temporary spike of WSS. WSS is lowered to 0.9 Pa (left) periodically at each 10 hour. The artery adapts this temporary change of WSS resulting no transmigration (middle) and hence no plaque growth (right).
(TIF)

S4 Fig Comparing low WSS and transmigration position: Represents the overlapping of low WSS zone (from CFD) and transmigration positions (form ABM). The endothelium is activated only adjacent to the plaque. A) At 33% of cardiac cycle the blood flow is very small (Fig 3A) and so the WSS is also very small throughout whole artery. Hence the leukocytes (generated only near plaque) transmigration occurs from almost everywhere (red dots). B) Likewise at 6% of cardiac cycle blood flow is very high and so the WSS. Hence few leukocytes transmigrated.
(TIF)

S5 Fig ABM-CFD model is initially more sensitivity to IL-1β than TNF-α: A) Represents the highest concentration of IL-1β or TNF-α in an EC patch over a 50 day ABM-CFD simulation. The concentration is < 0.04U/ml during the entire simulation. B) Shows the ABM rules for probability of neutrophil adhesion as a function of TNF-α and IL-1β concentration. Therefore, over a 50 day simulation, neutrophil adhesion is primarily due to IL-1β.
(TIF)

S6 Fig ABM-CFD model exhibits minimal stochasticity in regards to the predicted number of cells in the wall with time: The figure represents the average number of each leukocyte in the artery wall over a 130 day simulation. Shaded areas represent the standard deviation from three repetitions. The standard deviation is never above 220 in all cases. Indicating minimal stochasticity in the model despite having probabilistic functions.
(TIF)

S7 Fig **Spatial distribution of cells in the artery wall:** Longitudinal (A) and corresponding transverse (B) cross sections in a 7-month simulation of atherosclerosis, where endothelial cells, arterial cells, neutrophils and monocytes are represented by green diamonds, red circles, blue crosses and black dots, respectively. All other cells in the wall (lymphocytes, M1, M2 and foam cell) are represented in yellow. Neutrophils and monocytes coalesce towards the cap, shoulder regions, and adventitia but to a lesser extent at the central region of plaque.
(TIF)

S8 Fig 100μm patch size is reasonable given the sensitivity in change cytokine concentration does not change much: Schematic presentation of patches and the cytokine concentration on the corresponding patches. Obviously, there is not much change in the neighbor patches. So considering same cytokine concentration throughout a patch is reasonable.
(TIF)
