# Supplementary figures and images for: An agent-based model of leukocyte transendothelial migration during atherogenesis
Source: PLoS Comput Biol. 2017 May 25;13(5):e1005523. doi: 10.1371/journal.pcbi.1005523 (PMC5444619; doi:10.1371/journal.pcbi.1005523)

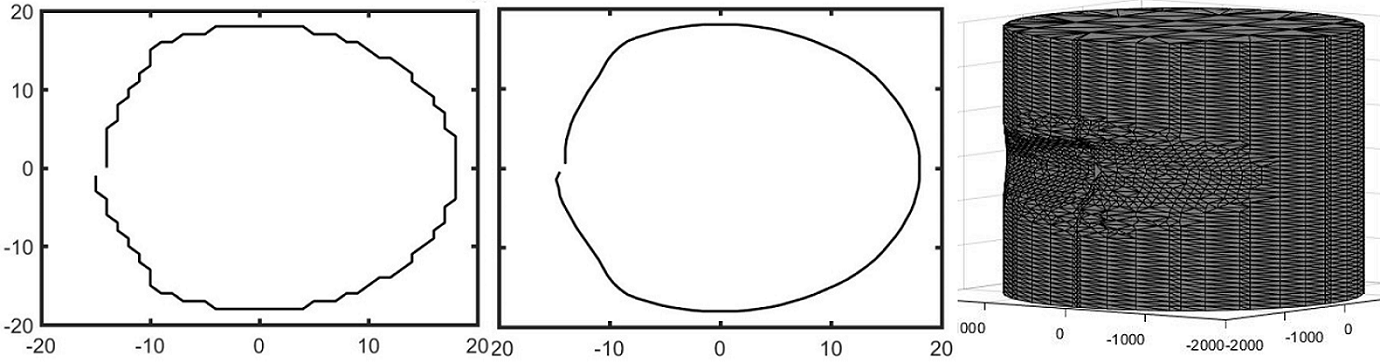

Supplement: S1 Fig — Since agents in ABM are at centroid of patches, the inner layer is a saw-toothed line. Middle: Smooth surface of the inner layer. Right: STL file generated in MATLAB. (TIF) [file pcbi.1005523.s002.tif]

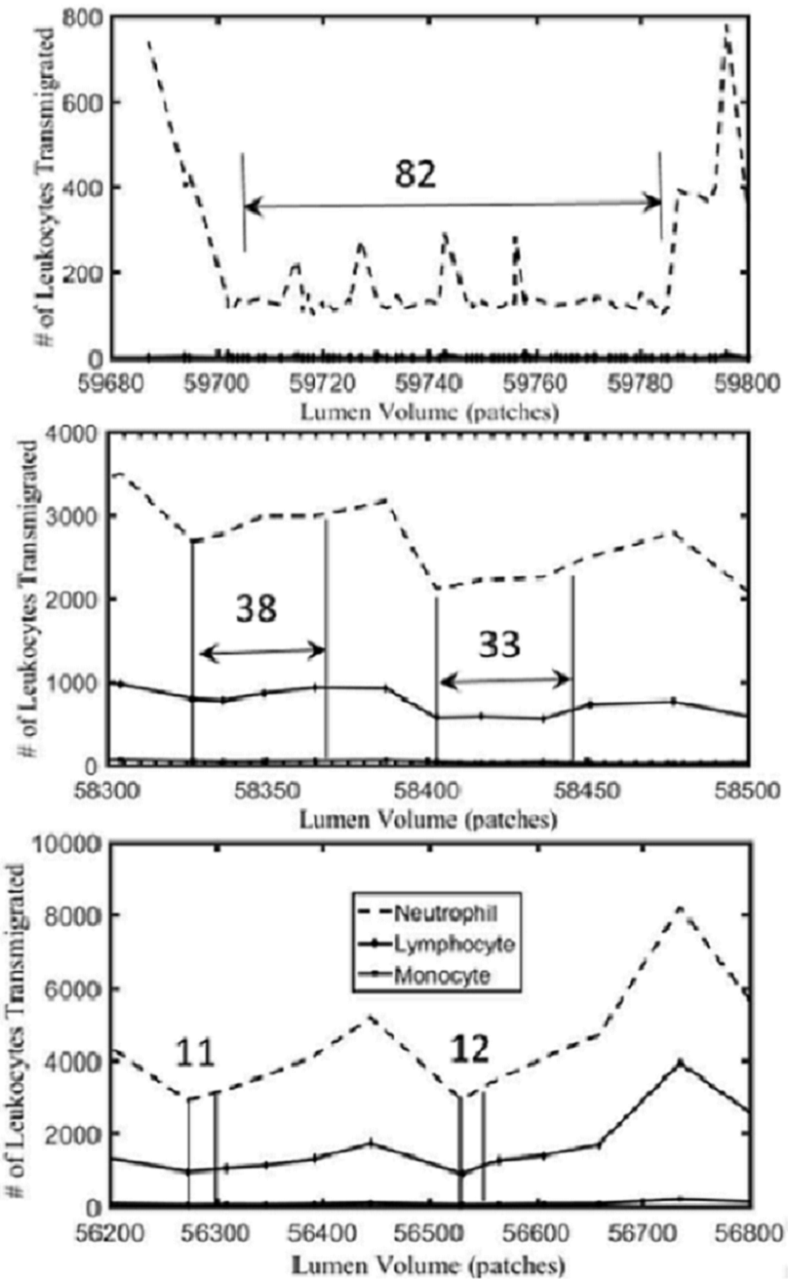

Supplement: S2 Fig — It is found that for small (A), medium (B) and big (C) spherical plaques, up to change of lumen patches of 80, 35, 10, respectively. TEM is almost constant. Hence WSS update is necessary after change of 80, 35, and 120, respectively. (TIF) [file pcbi.1005523.s003.tif]

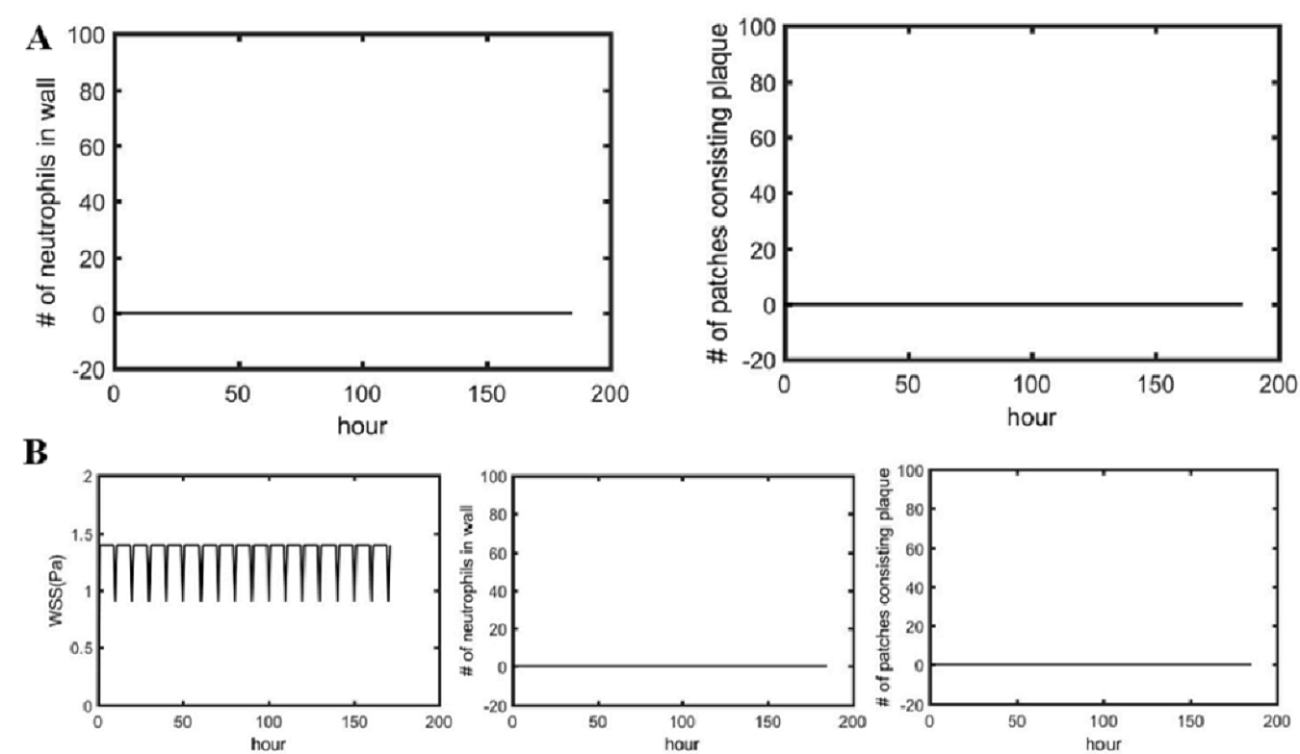

Supplement: S3 Fig — ABM ensures homeostatic and stability under temporary spike: (A) the model is run with normal WSS (1.4 Pa) and inactivated endothelium. As expected no transmigration (left) as well as no plaque growth (right) is overserved. (B) An example result of the model under temporary spike of WSS. WSS is lowered to 0.9 Pa (left) periodically at each 10 hour. The artery adapts this temporary change of WSS resulting no transmigration (middle) and hence no plaque growth (right). (TIF) [file pcbi.1005523.s004.tif]

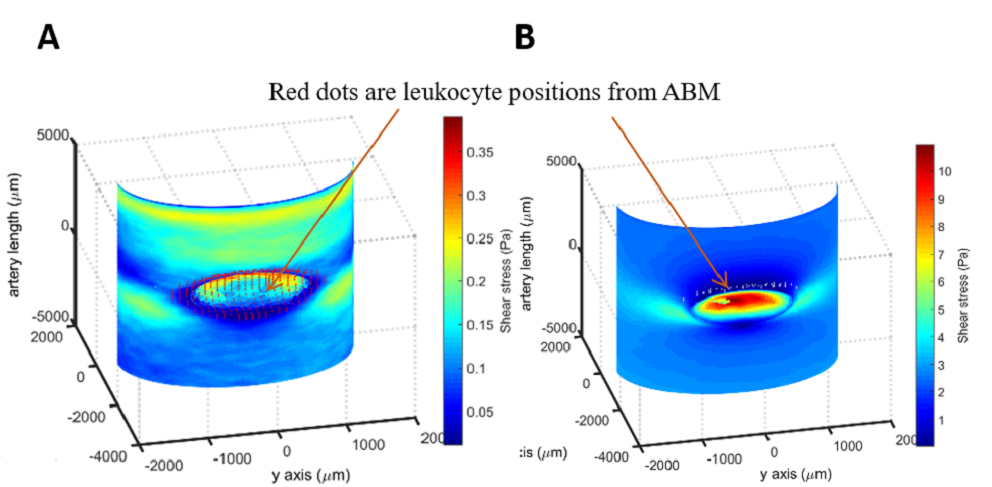

Supplement: S4 Fig — The endothelium is activated only adjacent to the plaque. A) At 33% of cardiac cycle the blood flow is very small (Fig 3A) and so the WSS is also very small throughout whole artery. Hence the leukocytes (generated only near plaque) transmigration occurs from almost everywhere (red dots). B) Likewise at 6% of cardiac cycle blood flow is very high and so the WSS. Hence few leukocytes transmigrated. (TIF) [file pcbi.1005523.s005.tif]

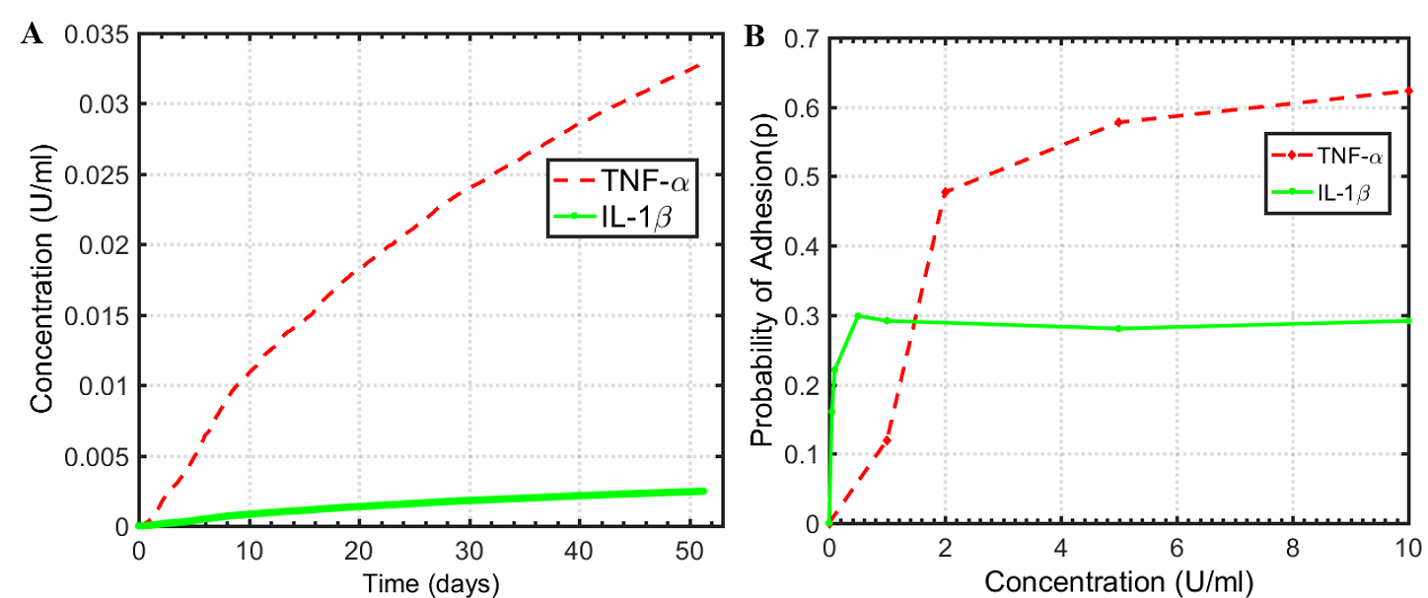

Supplement: S5 Fig — The concentration is < 0.04U/ml during the entire simulation. B) Shows the ABM rules for probability of neutrophil adhesion as a function of TNF-α and IL-1β concentration. Therefore, over a 50 day simulation, neutrophil adhesion is primarily due to IL-1β. (TIF) [file pcbi.1005523.s006.tif]

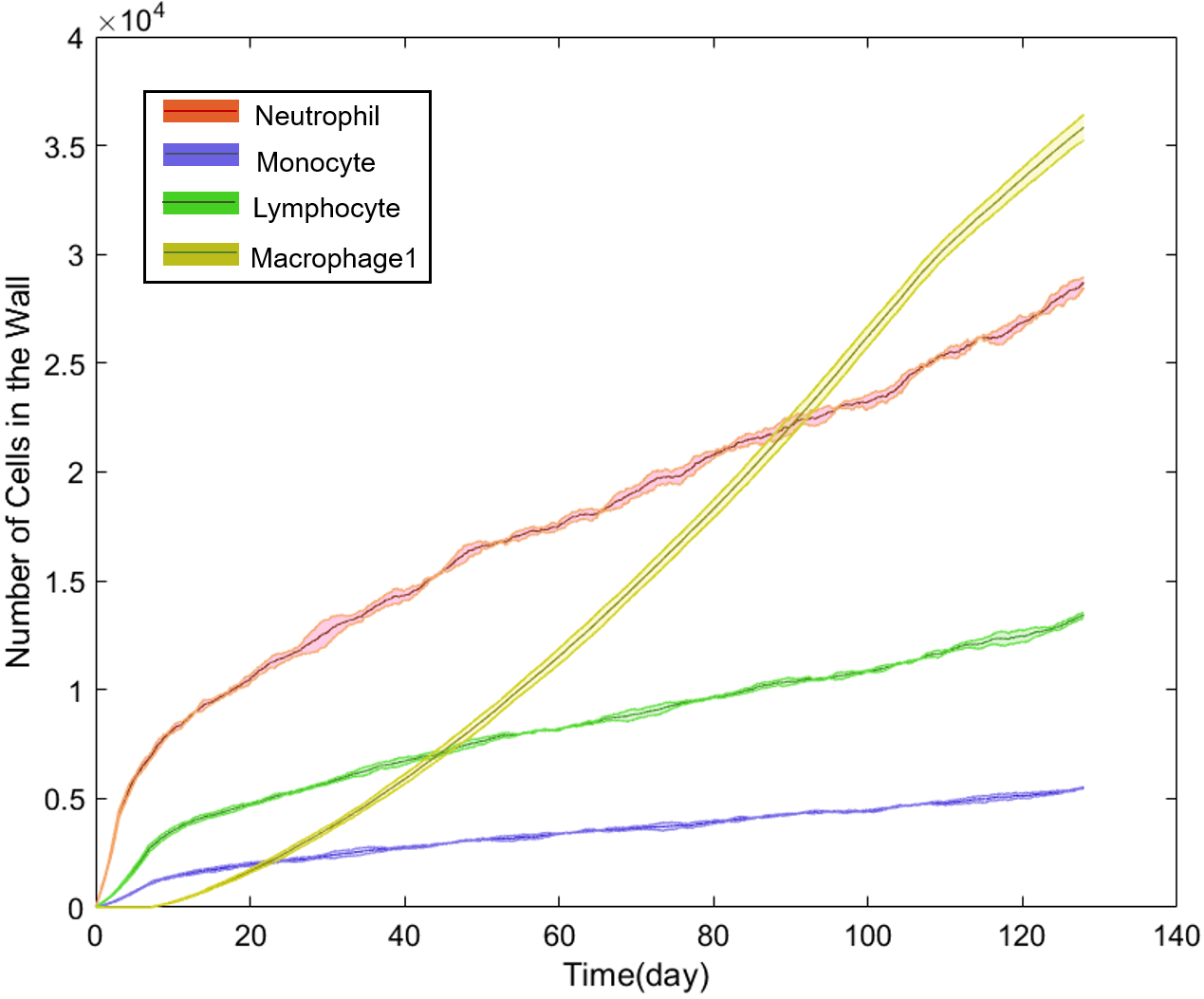

Supplement: S6 Fig — Shaded areas represent the standard deviation from three repetitions. The standard deviation is never above 220 in all cases. Indicating minimal stochasticity in the model despite having probabilistic functions. (TIF) [file pcbi.1005523.s007.tif]

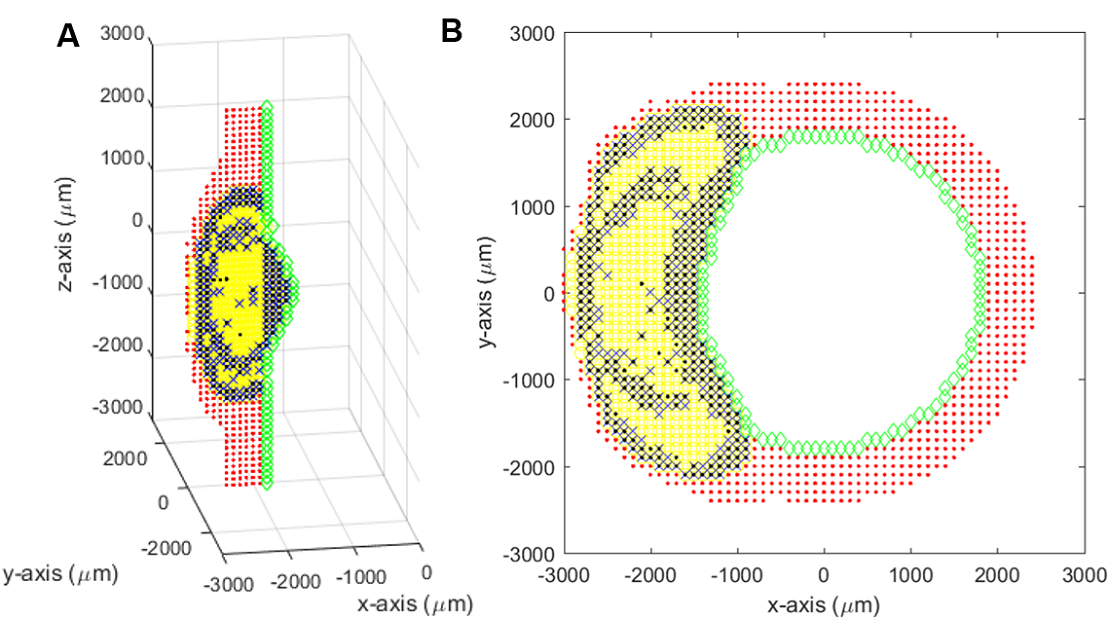

Supplement: S7 Fig — Spatial distribution of cells in the artery wall: Longitudinal (A) and corresponding transverse (B) cross sections in a 7-month simulation of atherosclerosis, where endothelial cells, arterial cells, neutrophils and monocytes are represented by green diamonds, red circles, blue crosses and black dots, respectively. All other cells in the wall (lymphocytes, M1, M2 and foam cell) are represented in yellow. Neutrophils and monocytes coalesce towards the cap, shoulder regions, and adventitia but to a lesser extent at the central region of plaque. (TIF) [file pcbi.1005523.s008.tif]

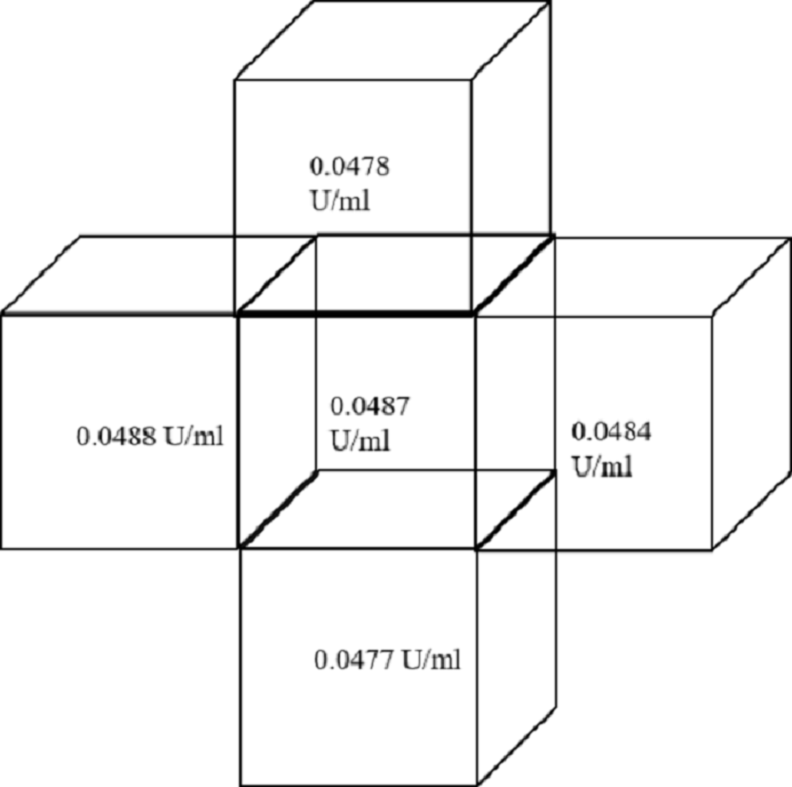

Supplement: S8 Fig — Obviously, there is not much change in the neighbor patches. So considering same cytokine concentration throughout a patch is reasonable. (TIF) [file pcbi.1005523.s009.tif]
